# Integration of HIV and cervical cancer screening perceptions and preferences of communities in Uganda

**DOI:** 10.1186/s12905-015-0183-4

**Published:** 2015-03-11

**Authors:** Edward Kumakech, Sören Andersson, Henry Wabinga, Vanja Berggren

**Affiliations:** School of Health and Medical Sciences, Örebro University, 701 82 Örebro, Sweden; Department of Pathology, Makerere University College of Health Sciences, PO Box 7072, Kampala, Uganda; Department of Laboratory Medicine, Örebro University Hospital, 703 62 Örebro, Sweden; Department of Health Sciences, Lund University, 221 00 Lund, Sweden

**Keywords:** HIV, Cervical cancer, Integration, Perceptions, Uganda, Screening

## Abstract

**Background:**

Despite the fact that HIV-positive women carry an increased risk of developing cervical cancer (CC) in comparison with HIV-negative women, HIV and CC screening programs in many developing countries have remained unintegrated. The objective of this study is to explore perceptions and preferences of community members in Uganda, including women, men, and village health teams, regarding the integration of HIV and CC screening services in a single-visit approach.

**Methods:**

This qualitative study was conducted in three districts in Uganda. Data were collected through focus group discussions with women and village health teams, and individual interviews with men. Respondents were purposely selected from among those linked to three CC clinics in the three districts. The content analysis method was used to analyze the data.

**Results:**

Three themes emerged from the data, namely appreciating the benefits of integration, worrying about the challenges of integration, and preferences for integration. The women endorsed the benefits. However, there were worries that integration would prolong the waiting time at the health facility and induce tiredness in both the healthcare providers and the women. There were also fears of being found positive for both HIV and CC and the consequences such as stress, self-isolation, and social conflicts. Participants, particularly the women, considered the challenges of screening integration to be manageable by, for example, taking a day off work to visit the hospital, delegating house chores to other family members, or taking a packed lunch on visiting the hospital.

**Conclusions:**

The community members in Uganda perceive the benefits of HIV and CC screening integration to outweigh the challenges, and expect that the challenges can be minimized or managed by the women. Therefore, when considering HIV and CC screening integration, it is important to not only recognize the benefits but also take into consideration the perceived challenges and preferences of community members.

## Background

Cervical cancer (CC) is the third commonest cancer among women globally in terms of 5-year prevalence, with an estimated 527,624 new cases and 265,672 deaths in 2012 [1]. Approximately 87% of the CC deaths occurred in less developed countries. In East Africa, CC is the most prevalent cancer and the leading cause of cancer-related deaths among women [1]. In Uganda the age-standardized incidence rate for CC was estimated at 44.4 per 100,000 women in 2012, which was one of the highest in the world [1].

HIV/AIDS is also a major cause of morbidity among adults globally, and more so in developing countries. The prevalence of HIV among women aged 15–49 years in Uganda increased from 7.5% in 2005 to 8.3% in 2011 [2]. In terms of burden, between 2007 and 2013 the estimated number of people living with HIV in Uganda increased from 1.2 million to 1.5 million, and 56% of these AIDS sufferers are women aged 15 years and older [2].

HIV infection has been associated with an increased risk of CC. In fact, an increasing body of literature indicates that HIV-positive women have an increased risk of developing CC in comparison with their HIV-negative counterparts [3-8]. In view these facts, the World Health Organization (WHO) recommends a more aggressive CC screening and treatment schedule for HIV-positive women than for HIV-negative women [9]. Such scheduling of CC screening according to HIV status of the women implies that women should be screened for HIV within CC programs. Furthermore, HIV and human papillomavirus (HPV), the cause of CC [10,11], are both sexually transmitted infections (STIs) and so share the same risk factors, such as early age at sexual debut, multiple sexual partners, and condom use [12-14]. Taken together, this should provide additional support for the integration of interventions for HIV and CC prevention to include information, education, communication, screening, and treatment in a single-visit approach.

Despite the aforementioned WHO recommendations and the available avenues for integration, in Uganda and many other developing countries HIV and CC prevention services continue to be implemented as stand-alone programs. For example, a study conducted in Nigeria among HIV-positive women attending post-HIV test counseling indicated that none of the respondents were informed about CC and its screening during the post-test counseling sessions [15]. Similarly, a study conducted in Uganda among healthcare providers (HCPs) and policymakers affirmed that much pessimism exists regarding the feasibility of HIV and CC screening integration [16]. As clinics remain non-integrated, almost all of the HIV care programs in Uganda do not offer CC screening services; hence, women attending HIV clinics miss CC screening opportunities despite the frequent visits they make to the HIV clinics for medical reviews and drug replenishment. Such missed opportunities for CC screening increase women’s risk of presenting late with advanced CC disease and a poor prognosis. Similarly, almost all CC screening programs in Uganda do not offer HIV screening services to women, so that these women risk receiving inappropriate schedules for CC screening. A less aggressive CC screening schedule for HIV-positive women also increases their risk of presenting late with advanced CC and a poor prognosis.

With regard to perceptions of the advantages of integration, recent studies concluded that there is a large potential health gain if HIV and CC prevention services are integrated [17,18]. In Zambia, where CC prevention clinics were co-located within the public health clinics offering HIV/AIDS care and treatment, multiple advantages were observed including resource and infrastructure sharing, availability of a wider range of health services for HIV-infected women, and opportunities for referral between the clinic systems and maximization of participation in both programs [19]. Similarly, in Kenya, were CC screening services were integrated into maternal child health (MCH) and family planning (FP) clinics, it was reported that a high proportion of women who visited the MCH-FP clinics for well-baby or FP services also benefited from CC and STI screening and treatment services in a single visit [20]. Moreover, in Nigeria, where women seeking reproductive health (RH) and/or HIV care services were bi-directionally referred to either HIV care or RH clinics co-situated within the same health facility, CC screening was highly accepted, and facilitated early detection and treatment of many cases of cervical pre-cancerous lesions among women [21,22].

However, literature about community members’ perceptions and preferences regarding the integration of HIV and CC screening remains sparse, particularly from Uganda. Diverse viewpoints from community members are critical considerations in developing effective health programs. Since 2010 the Uganda Ministry of Health has recognized the importance of considering the voices of the community members in health programs by establishing and stipulating roles for Village Health Teams (VHTs). According to the Uganda national health sector strategic plan 2010–2015, the responsibilities of the VHTs include, among others, identifying community health needs, mobilizing communities for health interventions, promoting health-seeking behaviors, maintaining registers of members of households and their health, birth, and death, and serving as a link between the community and formal HCPs [23]. By 2011, VHTs had been established in all communities in Uganda, each serving a maximum of 30 households. The selection of VHTs by communities in Uganda and the linkage they facilitate between the community and formal HCPs serves as an indication from the community members that they wish to have their voices heard in Ugandan health issues. The need to include the voices of the community members in health developments was re-echoed in a recent study conducted in Uganda among HCPs and policymakers regarding perceptions of HIV and CC screening integration, in which further studies on the perceptions among community members, especially the women, of HIV and CC screening integration were recommended [16].

Accordingly, the objective of this study was to explore the perceptions and preferences of community members in Uganda regarding HIV and CC screening integration in a single-visit approach. Information on community members’ perceptions and preferences, if made available, will inform the development of educational materials and employment aids that address misconceptions about integration, which if not confronted can potentially undermine the community members’ acceptability and utilization of the screening services within the integrated program.

## Methods

### Study design

A qualitative design was chosen for this study to allow for exploration of a wide range of views, opinions, and feelings of various members of the community regarding integration of HIV and CC screening services in a single-visit approach [24].

### Study sites

The study was conducted in three hospitals situated in three districts in Uganda, namely Kampala, Mbarara, and Ibanda. Each district had only one available CC clinic, located within one hospital of the district. Table 1 shows the characteristics of the participating hospitals and clinics. The participants from the three districts/CC clinics were diverse, providing perspectives from urban, semi-urban, and rural settings of Uganda. Moreover, the study sites provided perspectives from different levels of healthcare in Uganda, namely, the national referral hospital situated in the capital city of Kampala, the regional referral hospital situated in a semi-urban setting, and the district general hospital situated in a rural setting. One of the three participating hospitals was a private health facility while the other two were public health facilities.

**Table 1:** **Focus group discussions (FGDs) and individual interviews (IDIs) by respondent category**

**Participant category**	**Study site**			**Total**		
	**Kampala**	**Mbarara**	**Ibanda**	**FGDs**	**IDIs**	**Participants**
Women	2 FGs with a total of 13 participants	2 FGDs with a total of 12 participants	2 FGDs with a total of 15 participants	6	0	40
Village health teams (VHTs)	2 FGDs with a total of 11 participants	2 FGDs with a total of 12 participants	2 FGDs with a total of 13 participants	6	0	36
Men	4 IDIs	4 IDIs	4 IDIs		12	12
Total FGDs	4	4	4	12		
Total IDIs	4	4	4	12		
Total participants	28	28	32	88		

At the time of the study, all three participating hospitals had the experience of providing HIV and CC screening services as stand-alone programs for at least 3 years. The CC screening services they provided included health education talks, counseling, CC screening by visual inspection with 5% acetic acid solution, treatment of cervical precancerous lesions using cryotherapy, and referral for CC diagnosis, staging, and treatment. Moreover, none of the three clinics were implementing integrated delivery of HIV and CC screening services. HIV status of the women was therefore established through verbal reports from the women, and those who had never been tested for HIV were mostly presumed and treated as HIV-negative women.

### Selection criteria for study sites

The three hospitals were purposely selected to participate in the study. A hospital was eligible for selection if it was situated in any of the three study districts (Kampala, Mbarara, and Ibanda) and had clinics that provided stand-alone HIV and CC screening services. All three selected hospitals had only one CC clinic each, thus negating the need for further sampling within the selected hospital.

### Study respondents

The study involved three categories of respondents: women, men, and VHTs. These respondent categories reflect the three key members of any given community in Uganda. A VHT is the lowest, and thus the first, level of healthcare structure that operates at the village level (about 30 households). A VHT has no physical infrastructure but a committee comprising a maximum of five lay residents of a given village (of about 30 households) who are democratically selected by their fellow villagers. The VHT formation process is usually moderated by formal HCPs, and prospective members are selected to form the team based on their qualities such as trustworthiness, literacy, and availability and willingness to voluntarily mobilize their communities to take action on health issues. The VHTs from different villages report to, and are hence linked to, the same formal health facility nearest to their villages.

### Sample size

The sample for this study consisted of six women’s focus groups, another six VHT focus groups, and 12 men interviewed individually. The demographic characteristics of the participants are shown in Table 1. Being a qualitative study, the sample size of six women’s focus groups, six VHT focus groups, and 12 male interviews was determined by data saturation point, i.e., a point at which further sampling does not generate any new concepts or ideas about the phenomenon under investigation.

### Study field team and procedures

The study field team comprised the first author (E.K.), the last author (V.B.), and three clinic-level HCPs (one from each study site). The HCP at each study site assisted in providing the list of women and their male partners and the VHTs attending the clinic, and organizing the venues for the focus groups and interviews. The list of the CC clinic attendees were then used as sampling frames to select participants who fulfilled the eligibility criteria. All of the focus group discussions (FGDs) and individual interviews (IDIs) were conducted by the first and last authors (E.K. and V.B.).

### Sampling method for participants

From the list of women, male partners, and VHTs provided by each study site (sampling frame), participants from each study site were selected using a purposive sampling method [25]. Specifically for women’s focus groups and male interviews, the sampling procedure involved grouping the participants by their age, education, and occupation, and selecting from each of these sub-groups. The purposive sampling method allowed for selection of appropriate participants who had ever tested or received both HIV and CC screening services and thus had experience of undergoing both screening procedures. This method also allowed for selection of participants of diverse age, education, and occupation. Regarding VHT focus groups, the VHT’s structure and system whereby different VHTs from different villages are linked to the formal health facility nearest to their communities allowed us to obtain the list of members of VHTs linked to each study site. This list was then used to purposely select VHT members from different villages and gender to form the VHT focus groups for the study.

### Eligibility criteria for study respondents

Women, male partners, and VHT members were eligible to participate in the study if they were older than 18 years and had ever attended an HIV and/or CC screening clinic.

### Participants’ recruitment procedure

The researchers (E.K. and V.B.) visited each study site to develop a list of eligible participants for each study site (sampling frame). The list was developed from CC screening and treatment attendance registers, and VHT registers maintained by each clinic as a policy.

Based on the eligibility criteria, across all three study sites a total of 98 participants comprising 45 women, 38 VHTs, and 15 men were invited to the study. The invites were sent by telephone. Of the invitees, 88 accepted, three refused to participate, and seven could not be reached despite attempts to trace them with the help of community leaders. The response rate was therefore equivalent to 89.8%. For those who refused to participate, the two major reasons for refusal were insufficient transport refund provided by the study and lack of time to participate in the study.

### Data collection method

Data were collected between February 2013 and January 2014. FGDs and IDIs were used to collect the data depending on the category of the respondents. Focus groups were conducted with women and VHTs, and individual interviews were held with male partners of the women. Focus groups represented an appropriate method for collecting data from the women and VHTs because such discussions can allow diverse feelings, views, and opinions to be gathered within a short time. IDIs were appropriate for collecting data from male partners because this method has greater potential of allowing participants to freely share sensitive personal opinions or views such as beliefs, attitudes, and prejudices, which are more likely to be withheld in focus groups. All of the FGDs and IDIs were conducted by the first and last authors (E.K. and V.B.) in English and then translated into the local dialect (Runyankore), and the participants’ responses were in turn translated into English. All FGDs and IDIs were conducted in a quiet environment outside the CC clinic, and recorded using an audio recording device. A separate sheet of paper (one for each FGD and IDI) was used to record the sociodemographic characteristics of each respondent. Respondents’ names were neither recorded nor written anywhere to ensure confidentiality. The FGDs and IDIs lasted for 90–120 minutes.

### Focus group/interview guide

The instrument used for data collection was a semi-structured focus group/interview guide. The guide was specifically developed for the study and had items aimed at eliciting respondents’ feelings, opinions, or views about integrated delivery of HIV and CC screening services to women in a single-visit approach. The items in the guide were constructed based on theory of change and were phrased in such a way to allow respondents to examine and share their perceptions of the four quality control criteria of theory of change, namely plausibility, feasibility, testability, and appropriate scope [26]. In addition, some of the key questions in the focus group/interview guide included: what were the respondent’s impressions of integration of HIV and CC screening services in single-visit approach; what could be the advantages and disadvantages of integration; and how feasible, acceptable, and appropriate would integration be in public and private health sectors in a low-income country such as Uganda.

### Data analysis

**Figure 1:**
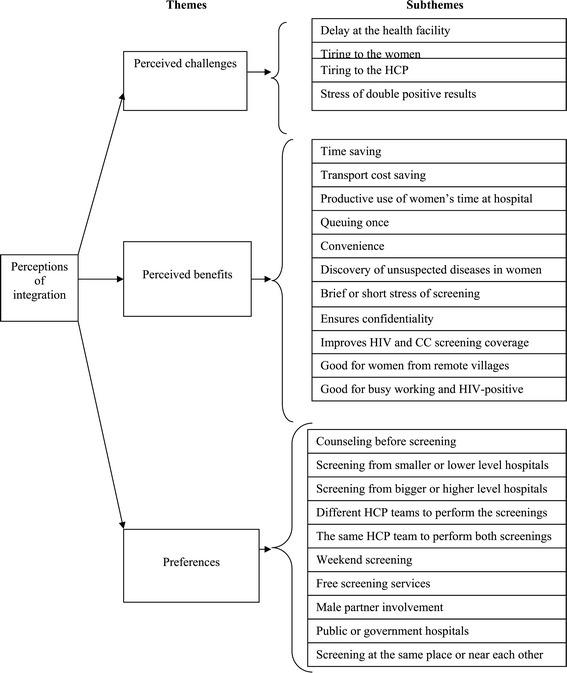
**Themes and subthemes.**

Data (from the audio recording) were first transcribed verbatim and then analyzed using the content analysis method [27], which allowed for exploration and synthesis of codes, themes, and categories. Figure 1 shows the code list together with the corresponding themes and categories that emerged from the data.

### Rigor of the research

The rigor of this qualitative study was ensured by paying attention to the credibility, trustworthiness, and transferability of the research [27]. The researcher’s prolonged engagement with the data during both the collection and analysis ensured the credibility of the data. To ensure trustworthiness of the study, several categories of respondents were utilized (also known as triangulation of data sources), and two researchers participated in the data collection and analysis (also known as triangulation of researchers) [25]. Furthermore, to ensure transferability of the study, the setting and context were explicitly described (see the Study sites section) [27].

### Ethical considerations

Ethical approval for the study was obtained from Institutional Review Board of the School of Biomedical Sciences, Makerere University College of Health Sciences. Informed consent was obtained from each respondent before inclusion in the study.

### Reporting guidelines

This paper was prepared in accordance with the RATS guidelines for reporting qualitative studies. RATS stands for relevance of the study question (R), appropriateness of the qualitative method (A), transparency of procedures (T), and soundness of the interpretive approach (S) [28]. This ensures compliance with internationally acceptable standards for reporting qualitative studies.

## Results

The community members’ perceptions of integration of HIV and CC screening fall under three themes, namely perceived benefits, challenges, and preferences (see Figure 1). Several subthemes, categories, or concepts emerged under each theme.

### Perceived benefits

#### Time saving

Across respondents’ categories and in all study settings, community members generally viewed integration of HIV and CC screening favorably. The integration attribute mentioned repeatedly in all focus groups and interviews was time saving, in relation to which some explained that it will save time in terms of not making a return journey for the other service, and that it would serve as a huge benefit and motivation for women to be patient and utilize the integrated services in a single visit.

*“It will save us time in terms of making a return journey for the other service”. –Women’s FGD, Kampala.*

*“It would save us time because we the women of nowadays are working for our families. Our husbands are no longer responsible. So if we can get a service like this which addresses two issues, then we shall not lose out in terms of time to go and work so that we earn money for a living”. – Women’s FGD, Kampala.*

#### Transport cost saving

While time saving emerged as a chief benefit for women and VHT focus groups across the study settings, for the men the advantage of an integrated HIV and CC screening was less about time saving than about transport cost saving.

*“Screening for both [HIV and CC] on the same day is very good and women will like it because people come from very far so it will help on paying for transport fares once instead of going and coming back which is double transport”. – Male interviewee, Ibanda.*

#### Productive use of women’s time at the hospital

Another perceived advantage that resonated with many women and VHT focus groups across the study settings was that integrated screening would allow productive use of women’s time at the hospital, which they would otherwise spend anyway traveling between stand-alone clinics.

*“Screening for both HIV and CC on the same day is good and women will like it because we always forgo one day to come to hospital so if we are screened for both it is good because you would have utilized your day well”. – VHT FGD, Ibanda.*

#### Queuing once

Across all study groups and settings, queuing once for both HIV and CC screening was noted as an advantage of the integrated approach. Women’s focus groups especially noted that queuing for one service for many hours only to be referred to another clinic by the HCPs, where one arrives and starts queuing afresh, can be discouraging. Therefore, an integrated approach demanding that women queue once only, however long it may be, is better.

*“What we don’t want is delaying us like yesterday where I spent too much time in the hospital since the women were so many there. So it is better to do everything at the same place or near each other. This makes the process faster*”. – *Women’s FGD, Kampala.*

#### Convenience

Women’s focus groups frequently remarked that integrated screening would not create the disruption of moving from one clinic to another seeking necessary services. Though mentioned occasionally by VHTs and male respondents, non-disturbance or the possibility to obtain all the screening services from one place was a key concept that emerged from women’s discussions. Women felt that integration could save them from the tiring travel between clinics looking for services, since a one-stop center would be available for all HIV and CC screening-related services, and they valued the convenience of being able to obtain all the services from one place.

*“The integration will be good because it does not require one to move from place to place. Everything is done in one place”. –Women’s FGD, Kampala.*

#### Discovery of unsuspected diseases

VHT focus groups across the study settings frequently remarked that integration of HIV and CC screening services would facilitate HCPs to discover unsuspected diseases in women. Early diagnosis of unsuspected diseases would in turn enable prompt initiation of treatment, leading to better health outcomes. An example of unsuspected diseases mentioned was vaginal Candida infection.

*“The integration is not bad because we have some illnesses that we do not know that we have. Integration will enable healthcare providers to catch and treat them promptly” – VHT FGD, Mbarara.*

*“Yes, I also think integration will enable healthcare providers to tell whether a woman is also suffering from other diseases of the private parts (reproductive system) for example Candida”. – VHT FGD, Mbarara.*

#### Brief or short stress or anxiety about screening

Women’s focus groups, particularly those from semi-urban and rural settings, remarked that HIV and CC screening integration would subject women to one-off stress or anxiety about screening, which with good counseling could be easily overcome, in contrast to the repeated stress or anxiety of HIV screening on one day and CC screening on another. In the women’s opinion the one-off stress or anxiety would be short-lived and, hence, advantageous, compared with the extended or prolonged stress or anxiety that would result from stand-alone screening programs.

“*If you know your CC status, you can at the same time know your HIV status and the advantage is that it prevents prolonged anxiety*”. – *Women’s FGD, Mbarara.*

*“When you test for both CC and HIV at once, the stress comes once, and when you are well counseled it’s okay”. – Women’s FGD, Kampala.*

#### Promotes confidentiality

Women’s focus groups frequently mentioned that integrated screening would promote confidentiality of health-related information because it would limit the number of HCP teams working with and accessing the women’s health information. Though not mentioned by VHTs and male respondents, the confinement of both HIV and CC screening information to limited teams of HCPs as a result of the integrated approach was a key concept that emerged from the women’s discussions. The women thought that the integrated screening model would prevent unnecessary access to their health-related information within the health system, and they valued this prospect of confidentiality for fear of stigmatization.

*“It also helps to keep confidentiality because whatever comes out of the screening will be between you and the health workers who performed both screening”. – Women’s FGD, Kampala.*

#### Improves HIV and CC screening coverage

Women’s focus groups and male respondents across the study settings frequently remarked that integrated screening would improve access to and, hence, coverage of both HIV and CC screening among women. The respondents thought that access to screening would be improved if every woman who came to any of the clinics for other health services were screened. Moreover, screening integration would improve HIV and CC screening coverage by bypassing delays in decision making, because women are less likely to waste time seeking additional permission from their male partners for each of the services within the integrated screening program if they find them there on offer after reaching the hospital, thus preventing any delay in decision making on the part of their male partners.

*“It will be good to integrate because even though you have come for another illness, they will take advantage of that and also screen you”. – Women’s FGD, Kampala.*

*“More women will benefit because if the woman leaves home after telling the husband that she is going for an HIV test and comes to understand that there is also screening for cancer, an issue that concerns her then she will also be screened for cervical cancer without necessitating her to ask permission from the husband which might take the man more time to be decided for her to come for the CC screening”. – Male Interviewee, Ibanda.*

#### Good for women from remote villages

Across study settings, women’s focus groups considered that women in remote villages would benefit more from integrated screening. Across study groups and settings, the target group unanimously identified as the best suited for integrated screening was village women, particularly those from remote areas who reside far away from health facilities. The respondents agreed that this particular group is best suited for an integrated screening program because their remoteness from the health facilities and low income level would not allow them to make several visits to distant health facilities for screening services.

*“We the village women will prefer this integration because it saves us on time and transport. Compared to the town women because they are near the services and can access it any time they want or when they are free”. – Women’s FGD, Ibanda.*

*“The women in the village will like it more because these women also have their work. They wake up very early in the morning and go to the garden so if it is a one day issue then they will welcome it because they will have to lose only one day and then the other days they resume their work”. – Women’s FGD, Kampala.*

#### Good for busy working women

According to the VHT focus groups and male interviews, busy working women, regardless of the distance between their residence and the health facilities, were thought to be an appropriate target and would benefit more from an integrated screening program. This view was based on their not having the time to make several journeys to the health facilities for screening services.

*“For my case if I go to the hospital the integration will help me because I have work to attend to and yet I have limited time. Women of now days have work to do. It is not like those days where women were just sited at home”. – VHT FGD, Kampala.*

#### Good for HIV-positive women

Across study groups and settings, it was frequently remarked that HIV-positive women are an appropriate target and would benefit more from the integrated HIV and CC screening strategy. However, this was not based on their knowledge of the science of HIV and CC interactions but rather on their observation that HCPs often urge HIV-positive women to attend CC screening in addition to their HIV/AIDS treatment.

*“Us HIV-positive women who are here right now we are the first to like the idea of integration. Even the health workers told us that it is good to go for the cervical cancer screening and you know your status such that if you don’t have then you are free and if you have it, then they will give you free treatment”. – Women’s FGD, Kampala.*

### Perceived challenges

#### Prolonged waiting time at the hospital

Across all study groups and settings, respondents spontaneously expressed concern over potential delay at the health facility as a result of integration. This fear of potential delay was further compounded among some respondents, especially women, who were aware of the fact that the existing stand-alone screening clinics are already crowded, with long queues. The respondents expressed fears that the delay at the health facility will be worsened by the unreliable means of transport for return journeys during evening or night hours, the lack of money to hire accommodation for an overnight stay, and the lack of caregivers for children left at home when staying away overnight.

*“What we don’t want is delaying us like yesterday where I spent too much time in the hospital since the women were so many there”. – Women’s FGD, Kampala.*

*“If the integration will take so long time that even the means of transport that we are to use has left us then we have to sleep over yet we have not planned for such conditions, this will not be good because in most cases you do not have anyone you know in town and at the same time you might have left your children with no one to take care of them”. – Women’s FGD, Mbarara.*

#### Tiring to the HCPs and the women

A number of respondents across study groups and settings also voiced (unprompted) their specific concern that HIV and CC screening integration would make the HCPs tired, while women in the queue will also become tired from a long waiting time. Boredom will also set in among the women waiting in the queue.

*“It wouldn’t be bad but its tiring on the side of a health worker. The health workers will get tired and will make the clients also tired”. – Women’s FGD, Mbarara.*

*“Boredom, if you wait for a long time in the queue”. – Women’s FGD, Kampala.*

#### Stress and social consequences of double positive results

Women’s focus groups spontaneously expressed their fears about the stress of discovering that they are double positive, i.e., positive for both HIV and CC at the same time, as a result of the integrated approach. This fear was more frequently expressed among the women from rural areas. Stress was thought to be an unavoidable consequence of the integrated screening. In particular, if the results turn out to be double positive, though not unusual in itself, this can be hazardous to the women as it can lead to automatic disclosure of their health status, marital conflict, violence, divorce, or even murder. Conversely, the fear of stress and the social consequences of double positive results never emerged from the VHT focus groups and male interviews.

*“One is stressed, after knowing I am HIV positive and they again find I am also CC positive, I get stress, after reaching home I am stressed, they would know my problem even if I don’t tell them”. – Women’s FGD, Ibanda.*

*“It’s not safe for the women, because after the husband knows that I am positive for both HIV and CC its very bad, he will say I am tired of you. And the husband will start barking at her, some husband may kill you or leave the family and go [divorce]. To avoid that some women fear to go and test, because of fear of how they will share the positive results with the husband and the rest of the family. So the women say even if it’s to die, let us die. We normally see these cases of negative reactions of husbands to positive health status of their women in our communities”. – Women’s FGD, Mbarara.*

### Preferences

#### Multi-skilled or transdisciplinary team to perform screening for both HIV and CC within the integrated program

Across all study settings, it was frequently remarked that women would prefer a multi-skilled or transdisciplinary HCP team to provide both HIV and CC screening within the integrated program. The respondents thought that a multi-skilled or transdisciplinary team would help in saving time, and enable the HCPs to get to know the clients better and provide better treatment. In addition, they remarked that all HCPs are sufficiently qualified to provide both HIV and CC screening.

*“The same HCP team will help in saving time and also the health workers get to know you better”. – Women’s FGD, Mbarara.*

#### Interspecialty or interdisciplinary team to perform the different screenings for HIV and CC within the integrated program

Although not discussed in the women’s focus groups from rural areas, it emerged particularly from the urban and semi-urban women’s focus groups that women would prefer interspecialty or interdisciplinary HCP teams to provide the different screenings for HIV and CC within the integrated program. The respondents thought that the use of interspecialty or interdisciplinary HCP teams would also help to speed up the screening process. Some remarked that the use of the interspecialty or interdisciplinary HCP teams would allow the different HCPs to practice the discipline of their specialty. Interestingly, it was also thought by some women that the use of interspecialty or interdisciplinary HCP teams to provide the different screenings within the integrated program would prevent one particular HCP from knowing the clients too well, which in the long run would minimize feelings of discomfort and fear of becoming a potential target for stigmatization and discrimination by HCPs in the integrated screening clinic.

*“It is better for different health workers to screen you for the different services because one health workers might know you so much so you might not be comfortable with that one knowing you so much so you would prefer another one to handle the other screening”. – Women’s FGD, Kampala.*

*“Because everyone has his/her specialty which he or she studied so the one screening for cancer should do that and the one screening for HIV should also do his part”. – Women’s FGD, Ibanda.*

#### Same place or nearby locations to house the integrated screening program

Across study settings, it was spontaneously remarked (unprovoked), especially from the women’s focus groups, that they would prefer the same location or places near each other to take part in the integrated program. The respondents thought that this would make the process faster and also minimize the inconvenience of moving from one clinic to another only to join another queue.

*“…. it is better to do everything at the same place or near each other. This makes the process faster”. – Male Interviewee, Mbarara.*

*“the problem we get is moving from here to there and after make a line for a service, so if the rooms are close to each other, then one will come from one room and after go to the other room to see the next doctor”. – Women’s FGD, Kampala.*

#### Village-level health centers for the integrated screening program

Across study settings, it was frequently remarked in the women’s focus groups that women would prefer the integrated screening to be scaled up to the village-level health centers. The respondents thought that the village health centers would help to address the distance and transport challenges, as women can easily reach such centers by foot or bicycle. The respondents also thought that scaling up integrated screening programs to village-level health centers will increase utilization and, hence, coverage of both HIV and CC screening services among women in rural areas and towns to an equal degree.

*“In the health centers that are in the villages. This would help us on issues concerning distance and transport”. – Women’s FGD, Mbarara.*

*“At some centers in the villages where one can be in position to even ride a bicycle and access the service”. – Male Interviewee, Ibanda.*

*“We would welcome the idea of integration more if they also bring us the services down in the villages and not leaving it in only to the town hospitals”. – Women’s FGD, Ibanda.*

#### Large hospitals for the integrated screening program

Although not heard of from the rural women’s focus groups, it was frequently remarked among the urban women’s focus groups that women would prefer large hospitals from which to obtain their integrated screening services. This preference was based on the belief that in the larger hospitals there would be enough rooms to screen many women simultaneously to save time, many HCPs, and the capacity for better management of the program.

*“It needs big hospitals so that there is enough space to examine many people.” – Women’s FGD, Ibanda.*

*“These services should be put in the big hospitals because the big hospitals have many health workers who can treat many illnesses.” – Women’s FGD, Kampala.*

*“Screening of theses illnesses should be put in the big hospitals because the clinics cannot manage.” – Women’s FGD, Mbarara.*

#### Public or government hospitals for the integrated screening program

Across study settings, the women’s focus groups spontaneously and frequently remarked that women would prefer public or government hospitals from which to obtain their integrated screening services. This preference was based on the high level of confidence the respondents put on public or government hospitals regarding their respect for people’s right to health and the provision of health services to all regardless of their socioeconomic background, unlike the private hospitals in Uganda.

*“These screenings should be in the government hospitals because ….. in the government hospitals they will work on you as a government person”. – Women’s FGD, Kampala.*

#### Integrated screening free of charge

Across study settings and groups, women’s focus groups frequently remarked that women would prefer to have access to integrated screening services free of charge. Several reasons were advanced for this, including the fact that CC disease is painless in the early stages so that people are less likely to pay for its screening; poor women would therefore prefer to allocate their little money to curative services rather than preventive services such as integrated screening. Other arguments advanced to explain why the respondents prefer free integrated screening services were the high likelihood that the male partners of women would not give money for screening and that fee for screening plus treatment and transport expenses would make screening very expensive and unaffordable to many women.

*“With this disease (cervical cancer) someone will not be in pain so it will not be easy for someone to get money and pay if they have no pain and she is not bed ridden so if you put a fee on it, then the project will not move well”. – Women’s FGD, Kampala.*

*“Not everyone will use this service if a fee is put to it because if someone does not get sick to the level of being bed ridden, then the person will not mind the screening test. It will be very difficult for people if they put a fee for the service”. – VHT FGD, Kampala.*

*“For some women it might be difficult because their husbands cannot give them the 2000 Uganda shillings to go for a test when they are not in pain”. – Male Interviewee, Kampala.*

*“If cancer screening is for money then it means that the drugs will also be for money and yet someone has already spent on transport so people will say that they better die because they do not have all that money”. – Women’s FGD, Ibanda.*

#### Male partner’s involvement

Across study settings, women prefer their male partners to be involved in the integrated screening for HIV and CC. However, they expressed a degree of pessimism and some reservations about their male partner’s involvement, perhaps the most critical being the remarks from women’s focus group that the issue of CC does not concern men enough for them to be motivated and get involved. There were also remarks about the incapacity or impossibility for women on their own to convince their male partners to become involved in the screening without the help of HCPs. Some women thought that male partners would not get involved because of other reasons such as lack of education about the issue, mistrust or fear that their wives would be cheating on them, lack of money for transportation, and lack of counseling.

*“For us as ladies we shall like the integration but it does not concern the men. So I don’t think that they will like it because it does not concern them”. – Women’s FGD, Kampala.*

*“It is very hard for a woman to convince her husband to go with her for HIV and cervical cancer screening so the man will not be around”. – Male Interviewee, Ibanda.*

*“Some husband will refuse because they mistrust their wives, they would fear that she is lying to go and cheat with their boyfriends”. – Male Interviewee, Kampala.*

*“If the men are well counseled and they are told that their wives have to be screened and if found to be having CC, then it can be treatable. The men will have no problem with it actually he will even welcome the idea……since cancer is a disease that scares everyone because it has no rescue”. – VHT FGD, Kampala.*

#### Educating the community about the integrated screening program

Across study settings, women expressed their wish to know beforehand the likely waiting time required to obtain an integrated screening for HIV and CC in a single visit so that they could prepare a packed lunch, acquire money for overnight accommodation if the need were to arise, and arrange house helpers to take care of the children left at home.

*“We are not supposed to be stressed by integration if there were education and counseling. Counseling helps much. There is no anything other thing that can happen if there is counseling”. – Women’s FGD, Mbarara.*

#### Dedicating a full day to the integrated screening program

Across study settings, women argued that dedicating a full day to attending the integrated screening clinic would solve the problem of lengthy delay at the clinic. Of course, such a solution can only work for the women after delegating the house chores to other family members and taking leave from work, allowing for waiting time without worry at the clinic.

*“For us the day we set for hospital is taken as a day for hospital and it means I will not go to work and I will also leave all the house hold chores. If I leave home I inform them that I have gone to hospital so they do everything and even though I reach late, I find everything done and I just rest. So we shall like the integration because you have set aside that hospital day so you have to be patient and everything is done on that day that you have set for hospital”. – Women’s FGD, Kampala.*

*“We shall like this new program because when you leave home for hospital it means you have given the hospital the whole day so there is no point of feeling that you are delayed”. – Women’s FGD, Mbarara.*

#### Being patient

Across study settings, it was frequently remarked in the women’s focus groups that women can remain patient in the queue while waiting to receive all of the integrated screening services. This emerged from the women’s groups as another solution to the potential delay at the clinic.

*“You can be patient because you are already there and you have been brought by the problem so you have to solve it so that you can save on your transport coming there for the same service on another day”. – Women’s FGD, Mbarara.*

*“We are used to that kind of schedule because when you leave home to go to hospital, the time they release you after they have worked on you that is the time you consider as your departure time so you have to be patient and they work on you”. – Women’s FGD, Kampala.*

#### Coming prepared with packed lunch

It was also frequently remarked from the women’s groups that one of the ways of coping with the challenges of integrated screening programs, more especially the issue of long waiting times, is for women to come to the hospital with a packed lunch.

*“If we get to know that it takes the whole day then we shall come when we are prepared in terms of lunch”. – Women’s FGD, Kampala.*

## Discussion

The respondents, particularly women, perceived the potential of deriving many benefits from the integration of HIV and CC screening in a single-visit approach. The perceived benefits included time saving, transport cost saving, productive use of women’s time at the hospital, convenience of queuing once for both screenings, discovery of unsuspected diseases among women, and improved uptake and coverage of both HIV and CC screening among women. The aforementioned community’s perceived benefits of an integrated screening program for HIV and CC concur with the study by Mwanahamuntu et al. [19] among HCPs and policymakers in Zambia. This study confirms that various categories of respondents in Uganda (i.e., women, men, VHTs, HCPs, and policymakers) had favorable views and were more likely to welcome integration of HIV and CC screening programs.

Unique to this study was the respondents’ view that an integrated HIV and CC screening program can potentially promote confidentiality of women’s health information. This was probably based on the belief that within an integrated screening program the same or relatively few HCP teams will perform the screening and thus have access to the women’s health information, reducing the likelihood of leakage of health information to uninvolved people within and outside the health system. Despite the lack of literature in relation to this aspect, the respondents’ perceived benefit of confidentiality of health information could be an indication that women who distrust the existing stand-alone screening programs for HIV and CC for reasons of lack of confidentiality and fear of subsequent stigmatization may prefer to attend the integrated screening program.

That the respondents in this study expressed fears that screening integration would lead to some women obtaining double positive results, leading to stress and potential marital conflicts as well as other social consequences such as domestic violence, divorce, and murder, warrants some attention. Previous studies have associated HIV-positive results with some of the aforementioned undesirable social consequences [15,29]. Specifically, this finding concurs with a study conducted in Nigeria which found that HIV-positive women who were not willing to undergo CC screening in the future, despite counseling, expressed fear of a cancer diagnosis which they did not wish to add to the already huge burden of HIV they were carrying [15]. Nevertheless, if such undesirable social consequences occur, they are unlikely to be a result of integrated screening but a lack of or ineffective counseling.

This study further revealed that there were fears among the respondents that screening integration would worsen the already long waiting time at the health facility, and its consequences such as fatigue and boredom among both the HCPs and women. Long waiting times at health facilities is an old problem in understaffed health systems in developing countries such as Uganda [30], and requires an improvement in staffing levels to absorb the extra load created by integration. Alternatively, the adoption of rapid and batch CC screening technologies such as multiplex HPV DNA testing using CareHPV™ with a self-sampling option can potentially reduce the workload of HCPs in an integrated HIV and CC screening program [31].

Respondents thought that the challenges associated with screening integration are manageable with means within their reach, such as counseling, community education, male partner involvement, and allocating a full day to the hospital visit to receive the screening after taking a day off work and delegating household chores to other family members. The respondents also thought that the overall benefits of screening integration outweigh the challenges. This finding indicates not only the importance the respondents in low-resource countries such as Uganda attach to an integrated screening program, but also their willingness to do everything possible within their means to access and utilize the services offered. However, being a qualitative study, such favorable perceptions of the integrated HIV and CC screening program expressed by respondents may not necessarily be shared by respondents from other settings in Uganda, but rather warrants pilot testing of integration in various study settings to validate such favorable perceptions.

The finding from this study that almost all respondent groups across study settings prefer integrated HIV and CC screening services to be free of charge while others are willing to share the costs warrants some discussion. Theoretically, it is possible for government entities such as the Ministry of Health to offer free integrated screening services if the resources saved from integration such as shared space and labor outweigh the cost of screening. However, in reality this is unknown, as no literature exists regarding this issue. On the other hand, the willingness of some of the respondents to contribute to the cost of integrated HIV and CC screening services could imply that due consideration should be given to the income levels of women when planning financial mechanisms for an integrated HIV and CC screening program.

It was also found that the respondents expressed pessimism about male involvement in the integrated screening programs for various reasons such as lack of information and money. Non-involvement of men in integrated HIV and CC screening programs is an issue that is not unique to this study, but an old problem that has plagued other RH programs such as FP [32]. It requires not only community education interventions targeting men but also the adoption of family- or couple-centered healthcare delivery approaches that are attractive to men.

### Study limitations

The use of focus group and individual interview research methods in this study limits the generalizability of the findings, but being a qualitative study it was not intended to be generalizable [33]. Another limitation concerns the lack of key informant interviews of selected knowledgeable members of the women’s focus group to validate data obtained from the FGDs. This limitation was partly addressed by including VHT focus groups in the sample because members of the VHTs are considered to be knowledgeable members of the community (both women and men) who promote health-related actions at the community level. In view of these limitations, the study findings should be used with caution in unrelated contexts or settings.

## Conclusions

Respondents in the low-resource setting of Uganda have expressed favorable and welcoming opinions about the integration of HIV and CC screening services, based on its economic and social benefits outweighing the likely challenges. In addition, the respondents prefer male involvement and affordable—if not entirely free of charge—integrated HIV and CC screening programs. There is a need for a pilot study of integrated HIV and CC screening at centers in Uganda and other related settings to validate the findings of this study.

## Abbreviations

HIV: Human immunodeficiency virus
CC: Cervical cancer
HCP: Healthcare provider
WHO: World Health Organization
HPV: Human papillomavirus
STI: Sexually transmitted infection
AIDS: Acquired immune deficiency syndrome
MCH: Maternal child health
FP: Family planning
RH: Reproductive health
